# Predictive Preoperative Score of Prolonged Mechanical Ventilation After Coronary Artery Bypass Grafting

**DOI:** 10.3390/jcm15166402

**Published:** 2026-08-19

**Authors:** Alba López-Lede, Juan Bertó, Jose María Barrio, María Jesus Pérez-Granda, Ignacio Vasserot, Manuel Martínez-Sellés, Begoña Quintana-Villamandos, Javier Hortal

**Affiliations:** 1 Cardiac Surgery Postoperative Care Unit, Hospital General Universitario Gregorio Marañón, 28007 Madrid, Spain; 2 Department of Pulmonology, Clínica Universidad de Navarra, 28027 Madrid, Spain; 3 Clinical Microbiology and Infectious Diseases Department, Hospital General Universitario Gregorio Marañón, 28007 Madrid, Spain; 4 CIBER de Enfermedades Respiratorias-CIBERES (CB06/06/0058), 28029 Madrid, Spain; 5 Department of Nursing, School of Nursing, Physiotherapy and Podiatry, Universidad Complutense de Madrid, 28040 Madrid, Spain; 6 Cardiology Department, Hospital General Universitario Gregorio Marañón, Instituto de Investigación Sanitaria Gregorio Marañón, CIBERCV, 28007 Madrid, Spain; 7 School of Biomedical Sciencies and Health, Universidad Europea de Madrid, 28670 Madrid, Spain; 8 School of Medicine, Universidad Complutense de Madrid, 28040 Madrid, Spain; 9 Department of Pharmacology and Toxicology, Universidad Complutense de Madrid, 28040 Madrid, Spain

**Keywords:** prolonged mechanical ventilation, coronary artery bypass grafting, preoperative score, anemia, cardiopulmonary bypass, patient blood management

## Abstract

**Background and Objective:** Prolonged mechanical ventilation (PMV) after cardiac surgery is a common complication associated with increased morbidity, mortality and intensive care resource utilization. Our aim was to determine predictors of PMV > 48 h in patients undergoing coronary artery bypass grafting (CABG). **Methods:** This was a single-center retrospective observational study including adult patients who underwent CABG between January 2011 and December 2024. The primary outcome was PMV. **Results:** From 2083 patients, 241 had PMV (11.6%). Compared with patients without PMV, those with PMV had lower hemoglobin levels (12.6 ± 2.1 vs. 13.4 ± 1.9 g/dL, *p* < 0.001) and worse estimated Glomerular Filtration Rate (67.1 ± 26.9 vs. 78.1 ± 22.6 mL/min, *p* < 0.001). The model with preoperative variables predicted the risk of PMV (with an area under the receiver operating characteristic curve [AUROC] of 0.789). The prediction improved with the addition of intraoperative variables (AUROC 0.832) and with the further addition of early postoperative re-exploration for bleeding (AUROC 0.847). A simplified preoperative score showed good discrimination (AUROC 0.78; 95% CI 0.749–0.81) and stratified patients into clinically meaningful risk categories. Restricted cubic spline analysis showed a non-linear association between cardiopulmonary bypass duration and PMV, with risk increasing progressively after 120 min. **Conclusions:** PMV after CABG can be predicted using simple preoperative variables. This prediction has relevant implications for clinical management, intensive care resource allocation, and surgical scheduling. Preoperative anemia. Preoperative anemia was independently associated with PMV and represents a potentially modifiable perioperative risk factor.

## 1. Introduction

Prolonged mechanical ventilation (PMV) remains one of the most important complications after cardiac surgery and is associated with increased morbidity, mortality, intensive care unit (ICU) length of stay, hospital costs, and long-term functional impairment [1,2,3,4,5,6,7,8,9]. Despite advances in perioperative management and fast-track recovery protocols, a clinically relevant proportion of patients still require prolonged respiratory support following coronary artery bypass grafting (CABG) [1,6].

The reported incidence of PMV after CABG varies considerably across studies, largely because of differences in patient populations and PMV definitions, ranging from approximately 2% to 10% [6,10,11,12]. Several predictors have been consistently associated with PMV, including advanced age, chronic obstructive pulmonary disease (COPD), renal dysfunction, ventricular dysfunction, urgent surgery, prolonged cardiopulmonary bypass (CPB), perioperative transfusion, and postoperative hemodynamic instability [10,11,12,13,14,15,16,17,18,19]. Although contemporary cardiac surgery risk models have substantially improved perioperative risk stratification, they were primarily developed to predict mortality and major postoperative complications rather than prolonged mechanical ventilation after isolated CABG [20,21,22]. Consequently, currently available predictive models remain limited by heterogeneous definitions and frequently rely on static preoperative variables, despite postoperative respiratory failure being a dynamic process influenced by preoperative vulnerability, intraoperative physiological injury, and postoperative complications [19].

Contemporary cardiac surgery increasingly involves older and higher-risk patients with a greater burden of comorbidities and surgical complexity [1,19]. Consequently, perioperative risk assessment should not rely exclusively on baseline patient characteristics but should evolve as additional information becomes available throughout the perioperative course. Furthermore, potentially modifiable factors such as preoperative anemia, perioperative transfusion, CPB duration, and the use of off-pump techniques may influence postoperative respiratory outcomes [23,24]. Emerging evidence also suggests that CPB-related pulmonary injury may not increase linearly with exposure time and that clinically relevant thresholds may exist beyond which respiratory complications rise disproportionately [23,25,26].

Recent systematic reviews have highlighted the need for contemporary and clinically applicable PMV prediction models capable of integrating sequential perioperative information and supporting individualized patient management [6,19]. Therefore, the objectives of the present study were to identify independent predictors of PMV after CABG surgery, develop sequential preoperative, intraoperative, and postoperative prediction models, construct a simplified clinically applicable preoperative risk score, and evaluate the impact of CPB duration, perioperative transfusion, and off-pump CABG on postoperative respiratory outcomes.

## 2. Methods

This was a single-center retrospective observational study. Every adult patient (aged ≥18 years) undergoing coronary artery bypass grafting (CABG) at the Department of Cardiac Surgery at Hospital General Universitario Gregorio Marañón, between January 2011 and December 2024 was included. Data were retrieved from an institutional registry containing demographic, clinical, laboratory, and procedural variables.

**Figure 1:**
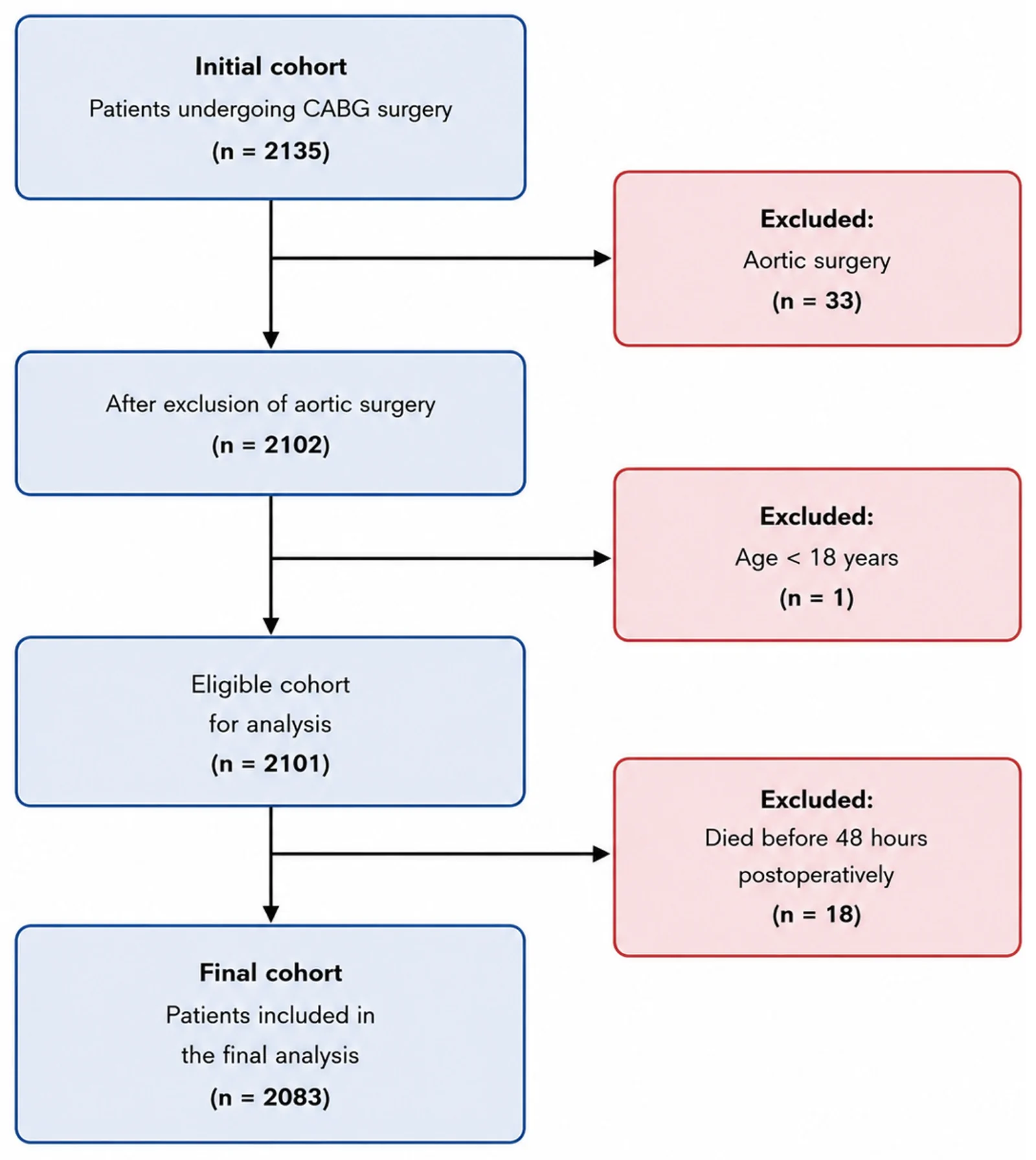
Flow diagram of patient selection. CABG—coronary artery bypass grafting.

Exclusion criteria were: (i) procedures involving the thoracic aorta; and (ii) patients who died within the first 48 h after surgery. A flow diagram of patient selection is provided in Figure 1.

The final study population consisted of 2083 patients, of whom 241 (11.6%) developed PMV.

### 2.1. Clinical Management, Variables and Definitions

All procedures were performed under general anesthesia, usually through median sternotomy. Cardiopulmonary bypass was used in most cases, although off-pump CABG (OPCAB) was performed in selected patients (17.3%). Postoperative care was standardized in the cardiac surgery intensive care unit according to institutional protocols based on Enhanced Recovery After Cardiac Surgery (ERAS) recommendations and the EACTS/EACTAIC cardiopulmonary bypass guidelines, including lung-protective mechanical ventilation, protocolized sedation and analgesia, daily assessment for weaning readiness, spontaneous breathing trials, early extubation whenever feasible, standardized hemodynamic management, and early physiotherapy and mobilization [27,28,29].

The primary outcome was PMV, defined as invasive mechanical ventilation delivered through an endotracheal tube for more than 48 h after surgery [20]. This threshold was selected because it coincides with the conventional temporal definition of ventilator-associated pneumonia (VAP), which is defined as pneumonia occurring after at least 48 h of endotracheal intubation [24,30]. We therefore considered this cut-off clinically meaningful, as it identifies patients exposed to a prolonged period of invasive ventilation and an increased risk of ventilator-related pulmonary complications. In patients requiring reintubation, the accumulated time of mechanical ventilation was considered. In patients who needed tracheostomy, the whole time of mechanical ventilation until decannulation was also considered. No patients were transferred to another institution or ward while still receiving invasive mechanical ventilation, as transfer from our ICU only occurs after successful weaning and extubation.

Candidate predictors were selected based on clinical relevance and previous literature reports and included demographic variables, comorbidities, cardiac status, laboratory parameters, and surgical characteristics [6,12,13,19]. Preoperative variables included age, haemoglobin, estimated glomerular filtration rate (eGFR), left ventricular ejection fraction (LVEF), pulmonary hypertension, COPD, urgent surgery, previous cardiac surgery, preoperative intra-aortic balloon pump (IABP), and surgical category. Continuous variables were categorized using clinically established thresholds to facilitate clinical interpretation and score construction. Surgical category was modeled as isolated off-pump CABG, isolated on-pump CABG, or combined CABG and valve surgery. Intraoperative variables included CPB time and intraoperative inotropic support with dobutamine (2.5–12.5 μg/kg/min) and adrenaline (0.01–0.1 μg/kg/min). The early postoperative model included re-exploration for bleeding occurring within the first 24 h. Perioperative transfusion was evaluated in a sensitivity analysis because it may represent an intermediate variable. LVEF dysfunction was categorized as mild/moderate (30–50%) and severe (<30%). Moderate pulmonary hypertension was defined as a systolic pulmonary artery pressure of 41–55 mmHg, and severe pulmonary hypertension as >55 mmHg, as estimated by preoperative echocardiography. Renal function was assessed using the estimated glomerular filtration rate (eGFR) calculated using the Chronic Kidney Disease Epidemiology Collaboration (CKD-EPI) equation and categorized as >60, 30–59, or <30 mL/min/1.73 m^2^. Urgent surgery referred to procedures performed during the same hospitalization that could not be postponed to a later admission.

### 2.2. Statistical Analysis

Continuous variables are presented as mean ± standard deviation or median [interquartile range], and categorical variables as counts and percentages. Group comparisons were performed using Student’s *t*-test, the Mann–Whitney U test for continuous variables, and the χ^2^ test for categorical variables and a *p*-value < 0.05 was considered statistically significant. Covariate balance was evaluated using standardized mean differences. Three multivariable logistic regression models were constructed: Model A, including preoperative variables; Model B, including preoperative and intraoperative variables; and Model C, including preoperative, intraoperative and early postoperative variables. Perioperative transfusion was not included in the primary models because of its potential role as an intermediate/mediating variable; a sensitivity analysis including transfusion was subsequently performed. Model performance was assessed by discrimination using AUROC, global accuracy using the Brier score, and relative fit using the Akaike information criterion (AIC). Calibration was evaluated across deciles of predicted risk. Candidate variables were selected a priori based on clinical relevance and previous literature. Model development and variable selection were performed according to established principles for contemporary cardiac surgery risk modelling [20,21,22]. Age was initially modeled using restricted cubic splines; as no relevant non-linearity was observed, it was included as a linear term in the final multivariable models. For the simplified clinical score, age was categorized to improve usability. Restricted cubic spline analysis was also used to explore the functional relationship between CPB duration and PMV, using 120 min as the reference value. Internal validation was performed using 1000 bootstrap resampling. Missing data were minimal and were handled using multiple imputation. The proportion of missing data was low for all variables, and no variable exceeded 5% missing values.

A simplified preoperative score was derived from the preoperative model by assigning integer points proportional to regression coefficients, retaining clinically relevant and stable predictors. The impact of off-pump CABG was assessed through multivariable adjustment and propensity score analysis. All analyses were performed with SPSS for Windows version 30.0.1.0 (IBM SPSS, Armonk, NY, USA) and Python version 3.11 (Python Software Foundation, Wilmington, DE, USA) for spline modelling and graphical outputs.

### 2.3. Ethical Considerations

The study was approved by the Institutional Research Ethics Committee on 18 November 2024, with a formal authorization issued on 3 December 2024. The Ethics Committee subsequently confirmed that an extension of the authorized study period did not require additional ethical approval because the study objectives and methodology remained unchanged. Patient enrollment for the present analysis was completed in December 2024.

### 2.4. Use of Generative Artificial Intelligence 

During the preparation of this manuscript, OpenAI ChatGPT (GPT-5.5) was used to assist with English language editing, improvement of grammar and writing style, refinement of the manuscript structure, preparation of figures generated. All outputs were critically reviewed, verified, and approved by the authors.

## 3. Results

### 3.1. Baseline Characteristics and PMV

A total of 2135 patients underwent CABG during the study period. After excluding patients undergoing aortic surgery (*n* = 33), those aged <18 years (*n* = 1), and patients who died within the first 48 h after surgery (*n* = 18), the final analytical cohort included 2083 patients (Figure 1).

PMV occurred in 241 patients (11.6%). Compared with patients without PMV, those with PMV were older and had a higher prevalence of anemia, renal dysfunction, ventricular dysfunction, atrial fibrillation/flutter, severe pulmonary hypertension, urgent surgery, previous cardiac surgery, and preoperative intra-aortic balloon pump use (Table 1).

### 3.2. Sequential Perioperative Models

In the full cohort, the preoperative model (Model A) showed a good discrimination with an apparent AUROC 0.789 (95% CI 0.757–0.818). After internal validation using 1000 bootstrap resamples, the optimism-corrected AUROC was 0.774 (95% CI 0.744–0.801), with a corrected calibration slope of 0.922 (95% CI 0.801–1.048). Complete model coefficients and internal validation results are provided in Supplementary Tables S3 and S4.

The strongest independent predictors in Model A were preoperative intra-aortic balloon pump use (OR 3.45; 95% CI 2.07–5.75), severe ventricular dysfunction (LVEF < 30%; OR 3.31; 95% CI 2.11–5.18), previous cardiac surgery (OR 2.9; 95% CI 1.54–5.44), combined CABG and valve surgery (OR 2.76; 95% CI 1.96–3.88), urgent surgery (OR 2.58; 95% CI 1.57–4.22), and severe renal dysfunction (eGFR < 30 mL/min/1.73 m^2^; OR 2.55; 95% CI 1.47–4.45). Preoperative anemia was also independently associated with PMV (OR 1.82; 95% CI 1.25–2.66) (Table A1).

Off-pump CABG was independently associated with a lower risk of PMV after multivariable adjustment (OR 0.58; 95% CI 0.36–0.94; *p* = 0.028).

The addition of intraoperative variables improved discrimination (Model B AUROC 0.832). In Model B, CPB duration (per 10 min), intraoperative dobutamine use, and intraoperative adrenaline use were independently associated with PMV, while major preoperative predictors remained clinically relevant (Table A2).

The inclusion of early postoperative re-exploration for bleeding resulted in a modest incremental improvement in discrimination (Model C AUROC 0.847), indicating that most of the predictive performance had already been achieved with the preoperative and intraoperative variables included in Model B. Re-exploration for bleeding was one of the strongest predictors in the final model (OR 4.13; 95% CI 2.71–6.28), together with intraoperative adrenaline use and preoperative IABP (Table A2, Model C).

**Figure 2:**
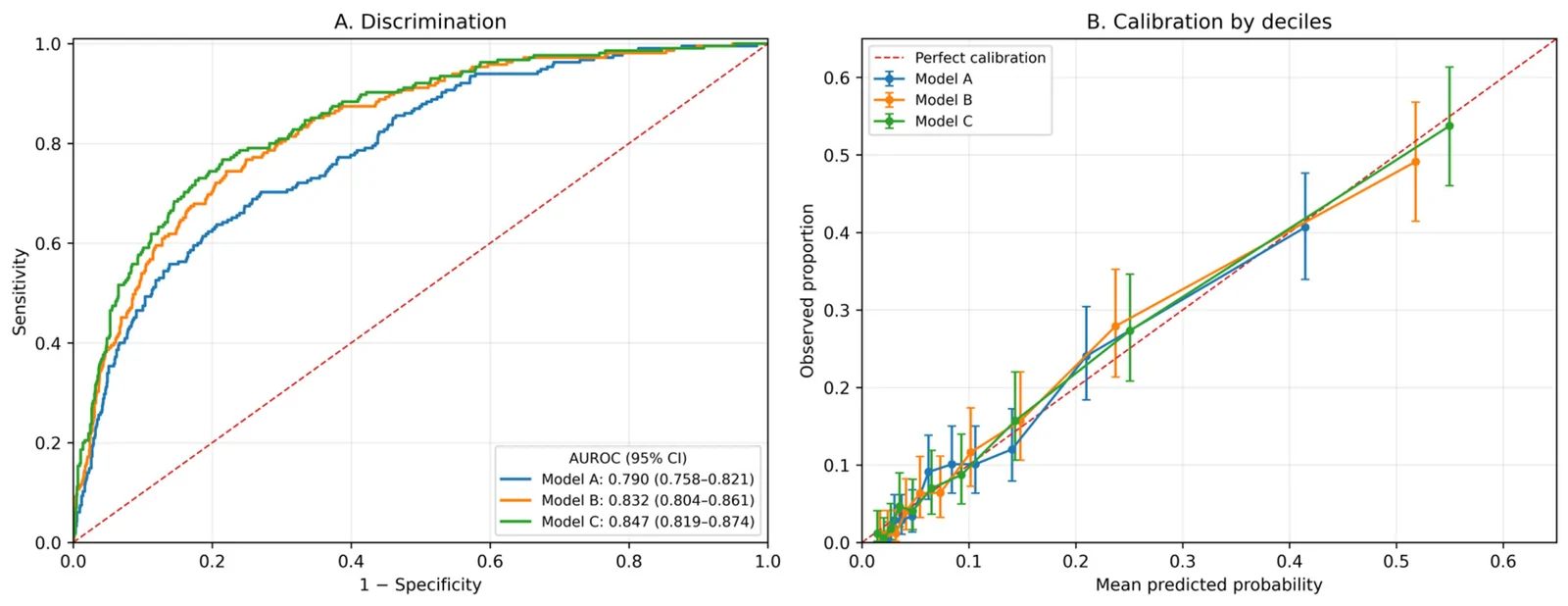
(**A**) ROC curves comparing Model A (preoperative), Model B (preoperative + intraoperative), and Model C (preoperative + intraoperative + re-exploration for bleeding) for prediction of prolonged mechanical ventilation. The red dashed diagonal line represents the line of no discrimination (AUROC = 0.5). (**B**) Calibration plots across deciles of predicted risk for Models A, B, and C. The red dashed diagonal line represents the line of perfect calibration. Panel (**A**): all models evaluated in the common CPB cohort (*n*= 1723; 215 PMV events). Panel (**B**): Model A evaluated in the full cohort (*n* = 2083); Models B and C in the CPB cohort (*n* = 1723). Calibration points represent deciles of predicted risk with 95% binomial confidence intervals.

**Figure 3:**
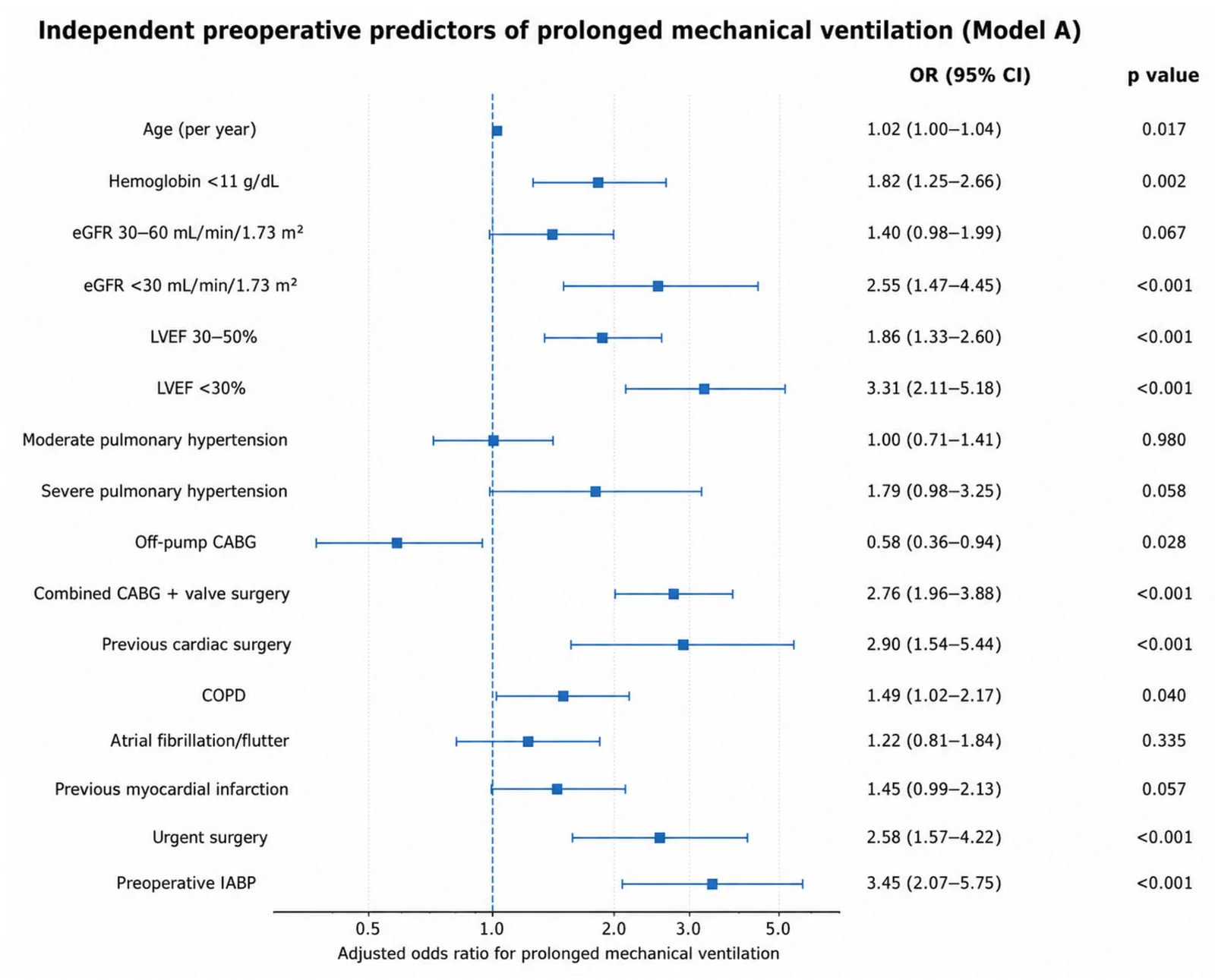
Forest plot of independent preoperative predictors of prolonged mechanical ventilation in Model A.

For comparative purposes, Models A, B, and C were also evaluated in the common CPB cohort (*n* = 1723), excluding patients undergoing off-pump CABG. Figure 2A compares the ROC curves of Models A, B and C. Figure 2B depicts calibration across deciles of predicted risk. Figure 3 summarizes the adjusted odds ratios and 95% confidence intervals for the independent predictors identified in the final preoperative model.

### 3.3. Non-Linear Association Between CPB Duration and PMV

Restricted cubic spline analysis demonstrated a significant non-linear association between CPB duration and PMV risk (*p* for non-linearity = 0.016).

**Figure 4:**
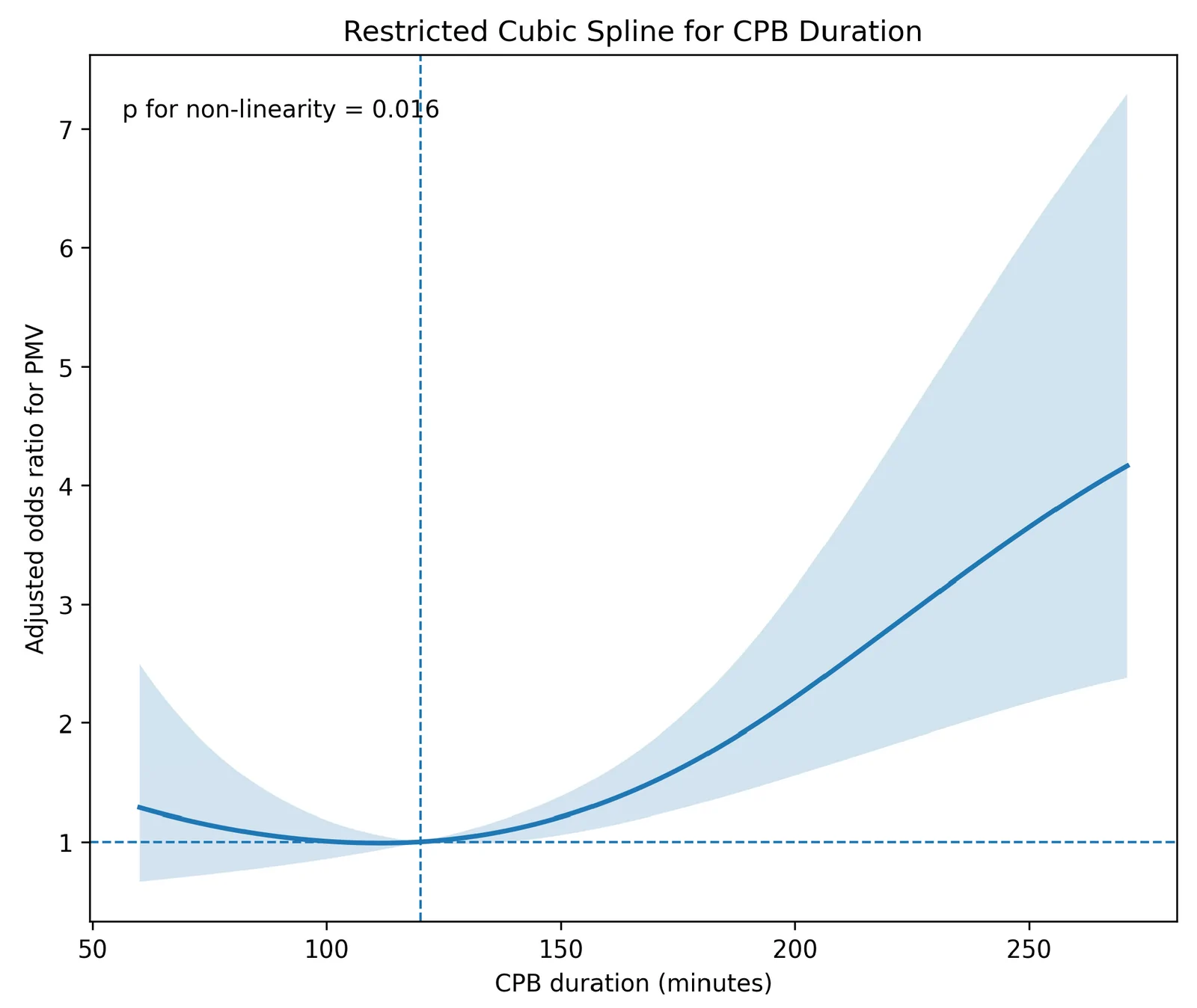
Restricted cubic spline analysis showing the association between cardiopulmonary bypass time and prolonged mechanical ventilation. The reference CPB duration was 120 min. The solid blue line represents the estimated odds ratio, and the shaded blue area represents the 95% confidence interval. The reference value (OR = 1) is indicated by the horizontal dashed line.

PMV risk remained relatively stable at shorter CPB durations but increased progressively beyond approximately 120 min of CPB exposure (Figure 4).

### 3.4. Simplified Preoperative Score

Reduced preoperative model comprising the variables included in the simplified score showed good discrimination, with an apparent AUROC of 0.783. After internal validation using 1000 bootstrap resamples, the optimism-corrected AUROC was 0.773, with a corrected calibration slope of 0.944 and a calibration intercept of 0.002. The simplified point-based score retained nearly all the discriminative performance of the reduced model, with an AUROC of 0.780 (95% CI 0.749–0.810). As expected, the simplified bedside score showed a slightly lower AUROC than the original multivariable preoperative model, reflecting the loss of information associated with categorization of continuous variables and conversion of regression coefficients into an integer-based scoring system.

The variables included in the score were age, hemoglobin < 11 g/dL, severe renal dysfunction, ventricular dysfunction, combined surgery, previous cardiac surgery, COPD, urgent surgery, and preoperative intra-aortic balloon pump use (Table 2).

The score stratified patients into clinically meaningful risk categories, with observed PMV risk increasing progressively from 2.9% in patients with 0–2 points to 44.3% in those with ≥9 points (Table 3). A score ≥ 6 identified a subgroup of patients with substantially increased PMV risk (>26.6%). Diagnostic performance metrics for different score thresholds are presented in Supplementary Table S5.

### 3.5. Perioperative Transfusion Sensitivity Analysis

Because perioperative transfusion may represent an intermediate variable in the causal pathway between baseline severity, surgical complexity and PMV, transfusion was not included in the primary multivariable models.

In a sensitivity analysis transfusion was added to Model B and emerged as one of the strongest independent predictors of PMV (OR 3.29 (95% CI 2.10–5.14; *p* < 0.001).

Inclusion of transfusion improved model discrimination (AUROC 0.832 vs. 0.844) and attenuated the effects of several perioperative predictors, particularly preoperative anaemia (43% attenuation) and CPB duration (16% attenuation), supporting a potential mediating role of transfusion in the pathway linking baseline vulnerability and operative complexity to prolonged mechanicl ventilation.

### 3.6. Off-Pump CABG

A total of 360 patients (17.3%) underwent off-pump CABG. Compared with patients treated with cardiopulmonary bypass, they had lower transfusion requirements, lower inotropic support, and a lower incidence of PMV (Table 4). Off-pump CABG was independently associated with a lower risk of PMV in the multivariable regression model (OR 0.58; 95% CI 0.36–0.94; *p* = 0.028). To further address potential selection bias, a dedicated propensity score analysis restricted to isolated CABG procedures was performed and confirmed this association (OR 0.41; 95% CI 0.25–0.65; *p* = 0.001). Covariate balances before and after Inverse Probability of treatment Weighting (IPTW) are shown in Supplementary Figure S1.

### 3.7. Comparison with EuroSCORE II

The simplified preoperative score showed an AUROC of 0.780 (95% CI, 0.749–0.810) for predicting prolonged mechanical ventilation, compared with 0.771 (95% CI, 0.740–0.801) for the estimated EuroSCORE II. The difference between the two AUROCs was not statistically significant according to DeLong’s test (*p* = 0.454). Although both models demonstrated similar discrimination, the proposed score was specifically developed to predict prolonged mechanical ventilation, whereas EuroSCORE II was originally designed to estimate operative mortality.

## 4. Discussion

In the present cohort of patients undergoing CABG surgery, PMV > 48 h occurred in approximately one out of every nine patients (11.6%) and was associated with substantial perioperative morbidity. This incidence is consistent with previous reports describing PMV rates ranging from approximately 2% to 12%, depending on the definition used, patient selection, and surgical complexity [5,6,7,8,9,10,11,12,13,14,15,16,17,18,19]. The present study identified robust preoperative, intraoperative, and postoperative predictors of PMV and demonstrated that predictive discrimination progressively improved after sequential perioperative variables were incorporated into dynamic multivariable models.

The preoperative model demonstrated good discrimination and identified several clinically plausible predictors of PMV, including severe ventricular dysfunction, advanced renal dysfunction, urgent surgery, previous cardiac surgery, combined CABG and valve procedures, COPD, anemia, and preoperative IABP use. Among these, IABP use, severe ventricular dysfunction, previous cardiac surgery, combined surgery, and severe renal dysfunction emerged as the strongest predictors. Collectively, these findings are consistent with previous studies and support the concept that PMV reflects the cumulative burden of impaired cardiopulmonary reserve, hemodynamic instability, and surgical complexity rather than an isolated respiratory complication [10,11,12,13,14,15,16,17,18].

Preoperative anemia emerged as a clinically relevant risk factor in our study. Approximately 13% of patients had hemoglobin levels <11 g/dL, and anemic patients required perioperative transfusion substantially more frequently than non-anemic patients (86.5% vs. 55.3%). Although our observational design does not allow causal inferences regarding the effect of anemia correction, these findings support careful preoperative assessment of hemoglobin levels as part of the overall risk evaluation. Previous studies have consistently associated both preoperative anemia and perioperative transfusion with increased postoperative morbidity, impaired oxygen delivery, inflammatory activation, and worse pulmonary outcomes after cardiac surgery [26,31,32,33,34,35,36]. Therefore, optimization of preoperative anemia within contemporary Patient Blood Management programs remains clinically appropriate to reduce transfusion requirements and improve perioperative care, although whether such strategies specifically reduce the risk of prolonged mechanical ventilation requires confirmation in prospective studies.

The incorporation of intraoperative variables significantly improved predictive discrimination. CPB duration, intraoperative dobutamine use, and intraoperative adrenaline administration independently predicted PMV in Model B. These findings support the concept that postoperative respiratory failure is not determined by baseline patient characteristics but also by the magnitude of intraoperative physiological injury.

Importantly, restricted cubic spline analysis demonstrated a significant non-linear association between CPB duration and PMV risk, with risk increasing progressively beyond approximately 120 min of CPB exposure. This observation is physiologically plausible and aligns with experimental and clinical evidence linking prolonged extracorporeal circulation with systemic inflammatory activation, pulmonary endothelial dysfunction, capillary leak, and impaired postoperative gas exchange [1,2,3,4,23,25,26]. From a clinical perspective, this finding suggests that efforts aimed at limiting CPB duration below critical exposure thresholds may potentially contribute to reducing postoperative respiratory dysfunction.

The postoperative model further improved predictive performance after incorporation of re-exploration for bleeding, which emerged as one of the strongest predictors of PMV. Re-exploration likely represents a composite marker integrating surgical bleeding, transfusion burden, hemodynamic instability, inflammatory activation, and prolonged sedation exposure. Previous studies have similarly demonstrated strong associations between postoperative bleeding complications and respiratory failure after cardiac surgery [32,33,34].

The role of perioperative transfusion deserves special consideration. Although transfusion remained independently associated with PMV in sensitivity analyses, it was intentionally excluded from the primary predictive models because it may represent an intermediate variable linking baseline patient characteristics, surgical complexity, perioperative bleeding, and postoperative pulmonary outcomes. Inclusion of transfusion resulted in only modest attenuation of the associations observed for the remaining predictors. While this pattern is compatible with the hypothesis that transfusion lies, at least in part, along the pathway between preoperative risk factors and PMV, the present analyses were not designed to formally evaluate mediation. Therefore, these findings should be interpreted as supportive of this biological hypothesis rather than as evidence of a mediation effect. This interpretation is biologically plausible given the inflammatory and immunomodulatory effects associated with blood product administration and transfusion-related lung injury [34,35,36,37].

The simplified preoperative score derived from Model A preserved most of the discriminative ability of the complete multivariable model and successfully stratified patients into clinically meaningful risk categories. Importantly, patients with scores ≥6 exhibited markedly increased PMV risk, supporting the potential utility of the score for perioperative risk stratification, patient counseling, ICU resource allocation, and targeted preventive strategies. Unlike previously published models that frequently incorporate postoperative variables or highly complex calculations, the present score relies exclusively on readily available preoperative variables, facilitating bedside applicability before surgery. Female sex was associated with PMV in univariable analysis but did not remain independently associated after multivariable adjustment.

Off-pump CABG was independently associated with a lower risk of PMV in the primary multivariable analysis, and this association remained significant after propensity score adjustment in the isolated CABG cohort. However, this finding should be interpreted in the context of potential selection bias, as the choice between on-pump and off-pump surgery is not random. The consistency of the findings after propensity score adjustment nevertheless supports the robustness of this association and is in line with previous randomized studies and meta-analyses suggesting improved pulmonary outcomes with off-pump techniques [23,24,25,26,30,38].

Unlike established cardiac surgical risk models, such as EuroSCORE II, which were developed to predict perioperative mortality and major morbidity, our score was specifically designed to estimate the risk of PMV. Therefore, it should be considered complementary rather than competitive with existing risk models.

These findings have important clinical implications. The preoperative score allows early risk stratification and may support ICU resource planning and surgical scheduling. However, perioperative risk should not be considered static. As intraoperative and early postoperative variables become available, risk estimation can be progressively refined, allowing a dynamic and stepwise approach to patient management. Early identification of high-risk patients may facilitate the implementation of targeted preventive strategies, including optimization of hemoglobin levels, lung-protective ventilation, early mobilization, and infection prevention measures. Such interventions may reduce the incidence of ventilator-associated pneumonia and improve postoperative outcomes [24,27,29,30,38].

The present study has several strengths. First, it integrates sequential perioperative information into dynamic predictive models rather than relying exclusively on static preoperative variables. Second, it evaluates the non-linear relationship between CPB duration and PMV using spline methodology. Third, it incorporates contemporary concepts of PBM, mediation analysis, and perioperative physiological progression. Finally, the simplified score provides a pragmatic and clinically applicable bedside tool.

Some limitations should also be acknowledged. This was a retrospective single-center study and therefore remains subject to selection bias and unmeasured confounding. In addition, changes in perioperative management during the study period may have influenced postoperative ventilation practices and ICU management strategies. PMV definitions also remain heterogeneous across the literature, potentially limiting direct comparisons between studies [5,6,19]. Finally, although transfusion was analyzed, the observational nature of the study precludes establishing causal relationships, particularly given its potential role as a mediator. External validation in independent cohorts will therefore be necessary before widespread clinical implementation of the proposed score and predictive models.

Nevertheless, the present findings suggest that PMV after CABG should not be interpreted as an isolated respiratory complication but rather as the final manifestation of cumulative perioperative physiological stress. Sequential perioperative modelling may therefore represent a clinically useful strategy to improve dynamic risk stratification, optimize perioperative management, and identify potentially modifiable factors associated with postoperative respiratory failure.

## 5. Conclusions

PMV after CABG is a common complication with significant clinical impact. We present a predictive preoperative scoring system that can be used as a practical and effective tool for identifying high-risk patients before surgery. By integrating demographic, clinical, laboratory and procedural variables, clinicians can stratify risk, optimize patient condition, and tailor perioperative management strategies. Preoperative anaemia represents a potentially modifiable risk factor and may be a target for optimization strategies. Risk estimation can be progressively refined as intraoperative and early postoperative variables become available.

## Figures and Tables

**Table 1:** Baseline characteristics according to prolonged mechanical ventilation (PMV).

Variable	Total (*n* = 2083)	No PMV (*n* = 1842)	PMV (*n* = 241)	*p*-Value
Age, years	66.7 ± 10.4	66.4 ± 10.4	68.8 ± 10.1	<0.001 *
Male sex	1637 (78.6%)	1466 (79.6%)	171 (71.0%)	0.003 *
Hypertension	1595 (76.6%)	1403 (76.2%)	192 (79.7%)	0.260
Diabetes mellitus	531 (25.5%)	471 (25.6%)	60 (24.9%)	0.883
COPD	304 (14.6%)	256 (13.9%)	48 (19.9%)	0.017 *
Atrial fibrillation/flutter	241 (11.6%)	192 (10.4%)	49 (20.3%)	<0.001 *
Moderate pulmonary hypertension	493 (23.7%)	418 (22.7%)	75 (31.1%)	0.005 *
Severe pulmonary hypertension	73 (3.5%)	50 (2.7%)	23 (9.5%)	<0.001 *
LVEF 30–50%	454 (21.8%)	380 (20.6%)	74 (30.7%)	<0.001 *
LVEF < 30%	167 (8.0%)	124 (6.7%)	43 (17.8%)	<0.001 *
Haemoglobin, g/dL	13.3 ± 1.9	13.4 ± 1.9	12.6 ± 2.1	<0.001 *
Haemoglobin < 11 g/dL	265 (12.7%)	202 (11.0%)	63 (26.1%)	<0.001 *
eGFR, mL/min/1.73 m^2^	76.8 ± 23.4	78.1 ± 22.6	67.1 ± 26.9	<0.001 *
eGFR 30–60 mL/min/1.73 m^2^	386 (18.5%)	319 (17.3%)	67 (27.8%)	<0.001 *
eGFR < 30 mL/min/1.73 m^2^	94 (4.5%)	67 (3.6%)	27 (11.2%)	<0.001 *
Isolated on-pump CABG	1129 (54.2%)	1038 (56.4%)	91 (37.8%)	<0.001 *
OPCAB	360 (17.3%)	334 (18.1%)	26 (10.8%)	0.006 *
Combined CABG + valve surgery	594 (28.5%)	470 (25.5%)	124 (51.5%)	<0.001 *
Urgent surgery	137 (6.6%)	99 (5.4%)	38 (15.8%)	<0.001 *
Previous cardiac surgery	64 (3.1%)	46 (2.5%)	18 (7.5%)	<0.001 *
Preoperative intra-aortic balloon pump	102 (4.9%)	65 (3.5%)	37 (15.4%)	<0.001 *

Values are mean ± standard deviation or *n* (%). * Statistically significant (*p* < 0.05). Abbreviations: CABG—coronary artery bypass grafting; COPD—chronic obstructive pulmonary disease; eGFR—estimated glomerular filtration rate; LVEF—left ventricular ejection fraction; PMV—prolonged mechanical ventilation.

**Table 2:** Simplified preoperative score for prolonged mechanical ventilation.

Variable	Points
Age < 60 years	0
Age 60–69 years	1
Age 70–79 years	1
Age ≥ 80 years	2
Haemoglobin < 11 g/dL	2
eGFR < 30 mL/min/1.73 m^2^	3
LVEF 30–50%	2
LVEF < 30%	3
Combined CABG and valve surgery	3
Previous cardiac surgery	3
Chronic obstructive pulmonary disease	1
Urgent surgery	3
Preoperative intra-aortic balloon pump	4

Abbreviations: LVEF—left ventricular ejection fraction; eGFR—estimated glomerular filtration rate.

**Table 3:** Observed risk according to categories of the simplified preoperative score.

Score Category	*n*	PMV Events	Observed PMV Risk
0–2	877	25	2.9%
3–5	783	83	10.6%
6–8	308	82	26.6%
≥9	115	51	44.3%

Abbreviations: PMV—prolonged mechanical ventilation.

**Table 4:** Comparison of preoperative, perioperative and outcome variables according to cardiopulmonary bypass use.

Variable	CPB (*n* = 1723)	OPCAB (*n* = 360)	*p*-Value
Age, years	66.2 ± 10.5	68.9 ± 9.8	<0.001 *
Atrial fibrillation/flutter	213 (12.4%)	28 (7.8%)	0.017 *
Severe pulmonary hypertension	68 (3.9%)	5 (1.4%)	0.025 *
LVEF < 30%	123 (7.1%)	44 (12.2%)	0.002 *
LVEF 30–50%	377 (21.9%)	77 (21.4%)	0.892
Hemoglobin, g/dL	13.4 ± 1.9	13.1 ± 2.1	0.003 *
Hemoglobin < 11 g/dL	196 (11.4%)	69 (19.2%)	<0.001 *
eGFR, mL/min/1.73 m^2^	77.7 ± 22.8	72.6 ± 25.3	<0.001 *
eGFR < 30 mL/min/1.73 m^2^	66 (3.8%)	28 (7.8%)	0.002 *
Urgent surgery	107 (6.2%)	30 (8.3%)	0.173
Previous cardiac surgery	57 (3.3%)	7 (1.9%)	0.232
Preoperative intra-aortic balloon pump	74 (4.3%)	28 (7.8%)	0.008 *
Perioperative transfusion	1105 (64.1%)	127 (35.3%)	<0.001 *
Intraoperative dobutamine	797 (46.3%)	61 (16.9%)	<0.001 *
Intraoperative epinephrine	99 (5.7%)	9 (2.5%)	0.017 *
PMV > 48 h	215 (12.5%)	26 (7.2%)	0.006 *
In-hospital mortality	79 (4.6%)	18 (5.0%)	0.840

* Statistically significant (*p* < 0.05). Abbreviations: PMV—prolonged mechanical ventilation; CPB—cardiopulmonary bypass; OPCAB—off-pump coronary artery bypass grafting.

## Data Availability

The data supporting the findings of this study are available from the corresponding author upon reasonable request.

## Supplementary Materials

The following supporting information can be downloaded at: https://www.mdpi.com/article/10.3390/jcm15166402/s1.

## Appendix A

**Table A1:** Independent predictors of the preoperative model.

Variable	Odds Ratio (OR)	95% CI	*p*-Value
Intercept	—	—	<0.001
Age (per year)	1.02	1.00–1.04	0.017 *
Hemoglobin < 11 g/dL	1.82	1.25–2.66	0.002 *
eGFR 30–60 mL/min/1.73 m^2^	1.40	0.98–1.99	0.067
eGFR < 30 mL/min/1.73 m^2^	2.55	1.47–4.45	<0.001 *
LVEF 30–50%	1.86	1.33–2.60	<0.001 *
LVEF < 30%	3.31	2.11–5.18	<0.001 *
Moderate pulmonary hypertension	1.00	0.71–1.41	0.980
Severe pulmonary hypertension	1.79	0.98–3.25	0.058
Off-pump CABG	0.58	0.36–0.94	0.028 *
Combined CABG + valve surgery	2.76	1.96–3.88	<0.001 *
Previous cardiac surgery	2.90	1.54–5.44	<0.001 *
COPD	1.49	1.02–2.17	0.040 *
Atrial fibrillation/flutter	1.22	0.81–1.84	0.335
Previous myocardial infarction	1.45	0.99–2.13	0.057
Urgent surgery	2.58	1.57–4.22	<0.001 *
Preoperative intra-aortic balloon pump	3.45	2.07–5.75	<0.001 *

* Statistically significant (*p* < 0.05). Abbreviations: OR—odds ratio; CI—confidence interval; LVEF—left ventricular ejection fraction; eGFR—estimated glomerular filtration rate; IABP—intra-aortic balloon pump.

**Table A2:** Model B intraoperative and Model C postoperative predictors.

Predictor	Model B OR (95% CI)	*p*	Model C OR (95% CI)	*p*
Age, per year	1.02 (1.00–1.04)	0.031	1.02 (1.00–1.04)	0.062
Haemoglobin < 11 g/dL	1.91 (1.25–2.93)	0.003	1.88 (1.22–2.91)	0.004 *
eGFR 30–60	1.57 (1.06–2.31)	0.023	1.59 (1.07–2.36)	0.021 *
eGFR < 30	2.77 (1.44–5.31)	0.002	2.83 (1.46–5.48)	0.002 *
LVEF 30–50%	1.50 (1.02–2.19)	0.038	1.52 (1.03–2.24)	0.037 *
LVEF < 30%	2.65 (1.58–4.45)	<0.001	2.66 (1.57–4.51)	<0.001 *
Moderate pulmonary hypertension	0.82 (0.56–1.22)	0.333	0.84 (0.56–1.26)	0.396
Severe pulmonary hypertension	1.48 (0.76–2.89)	0.247	1.40 (0.71–2.74)	0.327
Combined CABG + valve surgery	1.87 (1.28–2.73)	0.001	1.80 (1.23–2.65)	0.003 *
Previous cardiac surgery	2.23 (1.09–4.56)	0.027	2.33 (1.15–4.74)	0.019 *
COPD	1.49 (0.98–2.28)	0.062	1.44 (0.94–2.21)	0.098
Atrial fibrillation/flutter	1.00 (0.63–1.58)	1.000	1.11 (0.69–1.76)	0.670
Previous myocardial infarction	1.35 (0.88–2.08)	0.175	1.36 (0.88–2.12)	0.166
Urgent surgery	1.78 (1.00–3.16)	0.049	1.92 (1.07–3.44)	0.029 *
Preoperative IABP	3.36 (1.86–6.07)	<0.001	3.62 (1.98–6.60)	<0.001 *
CPB time (per 10 min)	1.06 (1.03–1.09)	<0.001	1.06 (1.03–1.09)	<0.001 *
Intraoperative dobutamine	1.97 (1.33–2.93)	<0.001	1.86 (1.24–2.77)	0.003 *
Intraoperative adrenaline	3.38 (2.05–5.57)	<0.001	3.25 (1.95–5.44)	<0.001 *
Re-exploration for bleeding	—	—	4.13 (2.71–6.28)	<0.001 *

* Statistically significant (*p* < 0.05). Abbreviations: OR—odds ratio; CI—confidence interval; CABG—coronary artery bypass grafting; CPB—cardiopulmonary bypass; COPD—chronic obstructive pulmonary disease; eGFR—estimated glomerular filtration rate; IABP—intra-aortic balloon pump; LVEF—left ventricular ejection fraction.
