# Supplementary material for: Predictive Preoperative Score of Prolonged Mechanical Ventilation After Coronary Artery Bypass Grafting
Source: J Clin Med. 2026 Aug 19;15(16):6402. doi: 10.3390/jcm15166402 (PMC13514348; doi:10.3390/jcm15166402)
Supplement: Supplementary file 1 [file jcm-15-06402-s001.zip › jcm-4415940-supplementary.pdf]

Supplementary material

1. Supplementary Table S1. Sensitivity analysis including the 18 patients who died within the first 48 hours after surgery.

**Variable**	**OR**	**95% CI**	***p*-value**
Age (per year)	1.02	1.01–1.04	0.0018*
Hemoglobin <11 g/dL	1.85	1.29–2.66	<0.001*
eGFR 30–60	1.40	0.99–1.98	0.054
eGFR <30	2.74	1.62–4.61	<0.001*
LVEF 30–50%	1.94	1.40–2.68	<0.001*
LVEF <30%	3.22	2.08–4.97	<0.001*
Severe pulmonary hypertension	1.75	0.99–3.08	0.053
Combined CABG + valve surgery	2.92	2.16–3.95	<0.001*
COPD	1.49	1.03–2.15	0.032*
Urgent surgery	3.14	1.99–4.95	<0.001*
Previous cardiac surgery	3.00	1.63–5.53	<0.001*
Preoperative intra-aortic balloon pump	3.29	2.00–5.40	<0.001*

2. Supplementary Table S2. Sensitivity analysis according to the study inclusion period.

**Variable**	**2011–2017 (*n*=1070)**	**2018–2024 (*n*=1013)**	**p-value**
Age, years	67.6 ± 10.1	65.7 ± 10.6	<0.001*
Male sex	824 (77.0%)	813 (80.3%)	0.071
Hypertension	839 (78.4%)	756 (74.6%)	0.042*
Diabetes mellitus	280 (26.2%)	251 (24.8%)	0.467
COPD	168 (15.7%)	136 (13.4%)	0.141
Atrial fibrillation/flutter	140 (13.1%)	101 (10.0%)	0.026*
Moderate pulmonary hypertension	313 (29.3%)	180 (17.8%)	<0.001*
Severe pulmonary hypertension	58 (5.4%)	15 (1.5%)	<0.001*
LVEF 30–50%	224 (20.9%)	230 (22.7%)	0.328
LVEF <30%	90 (8.4%)	77 (7.6%)	0.496
Hemoglobin, g/dL	13.3 ± 1.9	13.4 ± 1.9	0.289
Hemoglobin <11 g/dL	142 (13.3%)	123 (12.1%)	0.440
eGFR, mL/min/1.73 m^2	76.6 ± 23.0	77.0 ± 23.7	0.684
eGFR 30–60 mL/min/1.73 m^2	194 (18.1%)	192 (19.0%)	0.629
eGFR <30 mL/min/1.73 m^2	50 (4.7%)	44 (4.3%)	0.717
Off-pump CABG	171 (16.0%)	189 (18.7%)	0.106
Combined CABG + valve surgery	330 (30.8%)	271 (26.8%)	0.040*
Urgent surgery	24 (2.2%)	113 (11.2%)	<0.001*

Previous cardiac surgery	31 (2.9%)	33 (3.3%)	0.634
Preoperative intra-aortic balloon pump	40 (3.7%)	62 (6.1%)	0.012*
Prolonged mechanical ventilation	125 (11.7%)	116 (11.5%)	0.869

3. Supplementary Table S3. Complete multivariable preoperative logistic regression model (Model A).

**Predictor**	**β coefficient**	**Standard Error**	**Odds Ratio**	**95% CI**	***p*-value**
Intercept	-4.621	0.550	—	—	<0.001
Age (per year)	0.019	0.008	1.02	1.00–1.04	0.017*
Hemoglobin <11 g/dL	0.599	0.193	1.82	1.25–2.66	0.002*
eGFR 30–60 mL/min/1.73 m²	0.333	0.182	1.40	0.98–1.99	0.067
eGFR <30 mL/min/1.73 m²	0.937	0.283	2.55	1.47–4.45	<0.001*
LVEF 30–50%	0.619	0.171	1.86	1.33–2.60	<0.001*
LVEF <30%	1.196	0.228	3.31	2.11–5.18	<0.001*
Moderate pulmonary hypertension	-0.004	0.176	1.00	0.71–1.41	0.980
Severe pulmonary hypertension	0.580	0.306	1.79	0.98–3.25	0.058
Off-pump CABG	-0.540	0.246	0.58	0.36–0.94	0.028*
Combined CABG + valve surgery	1.015	0.173	2.76	1.96–3.88	<0.001*
Previous cardiac surgery	1.065	0.321	2.90	1.54–5.44	<0.001*
COPD	0.397	0.193	1.49	1.02–2.17	0.040*
Atrial fibrillation/flutter	0.201	0.208	1.22	0.81–1.84	0.335
Previous myocardial infarction	0.372	0.195	1.45	0.99–2.13	0.057
Urgent surgery	0.947	0.251	2.58	1.57–4.22	<0.001*
Preoperative intra-aortic balloon pump	1.238	0.261	3.45	2.07–5.75	<0.001*

4. Supplementary Table S4. Internal validation and calibration of the preoperative prediction model.

Parameter	Apparent performance	Optimism-corrected performance
AUROC	0.789 (95% CI 0.757–0.818)	0.774 (95% CI 0.744–0.801)
Mean optimism	—	0.015
Brier score	0.0886 (95% CI 0.0796–0.0983)	0.0915 (95% CI 0.0833–0.1004)
Calibration slope	1.000	0.922 (95% CI 0.801–1.048)
Calibration intercept	0.000	−0.130 (95% CI −0.370 to 0.142)
Calibration-in-the-large	0.000	0.003 (95% CI −0.137 to 0.150)
Bootstrap resamples	1000	1000

5. Supplementary Table S5. Sensitivity, specificity, and predictive values of the simplified preoperative risk score.

Positive threshold	Sensitivity (95% CI)	Specificity (95% CI)	PPV (95% CI)	NPV (95% CI)
Score ≥6	55.2% (48.9–61.3)	84.4% (82.6–86.0)	31.6% (27.3–36.2)	93.5% (92.2–94.6)
Score ≥9	21.2% (16.5–26.8)	96.7% (95.8–97.4)	45.5% (36.6–54.8)	90.4% (89.0–91.6)

6. Supplementary Figure S1. Propensity score weighting and post-adjustment covariate balance

IPTW propensity-score analysis in the full cohort

A. Treatment effect

Analysis	OR	95% CI	p-value
Crude	0.55	0.36–0.83	0.005
IPTW-adjusted	0.41	0.25–0.65	<0.001

Cohort: 2,083 patients
360 OPCAB; 1,723 non-OPCAB
241 PMV events

B. Weight and balance diagnostics

Parameter	Value
Stabilized weight range	0.278–3.503
1st–99th percentile	0.438–2.099
Effective sample size	1,871.6
Maximum absolute SMD before IPTW	0.268
Maximum absolute SMD after IPTW	0.064

All post-weighting absolute standardized
mean differences were below 0.10.

C. Covariate balance

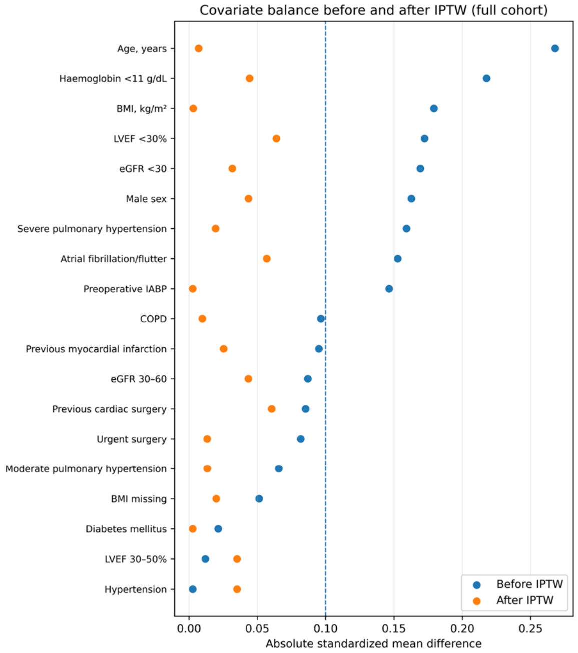

OPCAB versus non-OPCAB procedures. The dashed vertical line in panel C indicates the prespecified standardized mean difference threshold of 0.10.
